# Supplementary material for: Correlations Between Mammographic Breast Density and Outcomes After Neoadjuvant Chemotherapy in Patients with Locally Advanced Breast Cancer
Source: Cancers (Basel). 2025 Jul 1;17(13):2214. doi: 10.3390/cancers17132214 (PMC12249418; doi:10.3390/cancers17132214)
Supplement: Supplementary file 1 [file cancers-17-02214-s001.zip › cancers-3634625-supplementary.pdf]

| | | Non-obese | | | | Obese | | | | |
|---|---|---|---|---|---|---|---|---|---|---|
| | | low breast density | | high breast density | | low breast density | | high breast density | | p value |
| | | n | % | n | % | n | % | n | % | (obese/high MBD v other) |
| **Total** | | 31 | (26.3) | 46 | (39.0) | 26 | (22.3) | 15 | (12.7) | |
| **Age** | **Median** | 50 | | 46 | | 57 | | 42 | | |
| **T stage*** | **T1-2** | 15 | (48.4) | 21 | (48.8) | 10 | (43.5) | 1 | (7.1) | 0.002 |
| | **T3-4** | 16 | (51.6) | 22 | (51.2) | 13 | (56.5) | 13 | (92.9) | |
| **Lymph nodes** | **negative** | 18 | (60.0) | 24 | (57.1) | 9 | (37.5) | 5 | (33.3) | 0.08 |
| | **positive** | 12 | (40.0) | 18 | (42.9) | 15 | (62.5) | 10 | (66.7) | |
| **Grade** | **1-2** | 14 | (46.7) | 24 | (52.2) | 16 | (64.0) | 10 | (66.7) | 0.17 |
| | **3** | 16 | (53.3) | 22 | (47.8) | 9 | (36.0) | 5 | (33.3) | |
| **ER/PR** | **negative** | 10 | (32.3) | 17 | (37.0) | 8 | (30.8) | 4 | (26.7) | 0.12 |
| | **positive** | 21 | (67.7) | 29 | (63.0) | 18 | (69.2) | 11 | (73.3) | |
| **HER2** | **negative** | 24 | (75.0) | 34 | (73.9) | 16 | (61.5) | 10 | (66.7) | 0.36 |
| | **positive** | 8 | (25.0) | 12 | (26.1) | 10 | (38.5) | 5 | (33.3) | |

**Table S1:** Comparison of Patient and Tumour Demographics According to Composite Mammographic Breast Density and Body Mass Index Group
